# Pelvic Examination in Undergraduate Medical Education: A Scoping Review

**DOI:** 10.1111/tct.70475

**Published:** 2026-07-08

**Authors:** Carol Boutrous, Shweta Rao, Anna Harvey Bluemel, Megan E. L. Brown, Heidi Stelling

**Affiliations:** ^1^ School of Medicine Newcastle University Newcastle upon Tyne UK

**Keywords:** clinical skills, GTA'S, gynaecology teaching associate, pelvic examination, scoping review, teaching methods, undergraduate medial education

## Abstract

**Background:**

Pelvic examination is a core clinical skill in undergraduate medical education, requiring integration of technical competence with communication, consent and patient‐centred care. Despite its importance, opportunities to learn pelvic examination are variable, and educational approaches differ widely. Existing reviews have largely focused on short‐term learner outcomes rather than broader questions of implementation, cultural context and patient impact. This review aimed to map teaching methods used to teach pelvic examination, examine how effectiveness is defined and evaluated, and identify reported barriers and enablers to implementation.

**Method:**

A scoping review was conducted in accordance with Arksey and O′Malley's framework and prospectively registered with Open Science Framework. Database searches were undertaken in June 2024 and repeated in May 2026. Studies describing pelvic examination teaching for undergraduate medical students were included. Data were extracted and analysed using inductive qualitative content analysis. Outcomes were mapped to the Kirkpatrick model to enable comparison.

**Findings:**

Fifty‐five studies were included. Teaching approaches were grouped into received knowledge (e.g., lectures, videos and e‐learning) and experiential approaches (e.g., models, professional patients and clinical encounters). Most studies combined multiple modalities. Evaluation predominantly focused on learner‐centred outcomes at Kirkpatrick levels 1 and 2, particularly confidence and perceived competence. No studies assessed behavioural change or patient‐level outcomes. Implementation was influenced by resource availability, workforce pressures, organisational culture and equity considerations.

**Implications:**

Pelvic examination education requires balanced, feasible and culturally sensitive approaches that extend beyond short‐term learner outcomes. Future research should prioritise patient perspectives, long‐term behavioural change, inclusivity and cost‐effective implementation within real‐world clinical contexts.

## 1 Background

Pelvic examinations (speculum and bimanual vaginal examination) are core clinical skills in gynaecological practice. The ability of practitioners to perform them effectively is key to accurate diagnosis, referral, and treatment [1]. However, pelvic examination is intimate and potentially distressing, particularly if performed without sensitivity and competence [2]. Poor consent practices can cause psychological harm, and recent attention has heightened expectations around explicit consent and empathic communication during sensitive examinations [3]. Competence in pelvic examination extends beyond examination technique to include communication, dignity and consent.

Despite their importance, pelvic examination remains challenging to teach and learn [4]. Integrating technical manoeuvres with person‐centred communication, alongside the ethical tensions of learning “on” patients, creates an inconsistent and opportunity‐dependent training environment [5, 6]. Clinical exposure is also variable. In one study of clerkships, many students performed few pelvic examinations, and over half performed no bimanual examinations [6]. This has harmful consequences for patient care; 33%–74% of patients do not undergo a pelvic exam when it is clinically indicated [7]. Although barriers are multifaceted, graduates' lack of confidence, experience, and competence are recognised contributors [8]. Inconsistent clinical exposure, variable supervision, and the intimate nature of these examinations can leave students anxious, underprepared, and reluctant to engage in learning opportunities [4, 6], particularly when consent processes are unclear or teaching occurs in high‐pressure clinical environments [5]. These experiences may reinforce avoidance, limit opportunities for practice and feedback, and perpetuate gaps in confidence and identity formation around women's health [9].

*Inconsistent clinical exposure, variable supervision and the intimate nature of these examinations can leave students anxious, underprepared and reluctant to engage in learning opportunities*.

Learning pelvic examination extends beyond planned teaching sessions. Eraut distinguishes between codified (“received”) knowledge from the tacit, experiential knowledge developed through participation in clinical work [10]. While anatomical understanding or procedural steps may be introduced through formal teaching, other aspects of competence, for example, negotiating consent, are shaped through experience. Pelvic examination education, therefore, spans both curriculum‐led teaching and workplace‐based learning, where opportunity and workplace cultures influence knowledge application. Educational research has increasingly looked to evaluating approaches designed to support learning across these different settings.

A systematic review by Kirubarajan et al. [4] synthesised evidence regarding available teaching interventions, reporting outcomes focused on either student competence (often assessed objectively via OSCEs or ratings) or student comfort (commonly assessed subjectively through, e.g., self‐reported anxiety, confidence, or satisfaction). This review demonstrates that multiple teaching modalities—particularly programmes involving Gynaecological Teaching Associates (GTAs)—can improve learner outcomes. However, the focus of this review also reflects medical education's dominant focus on outcomes as proximal, learner‐centred endpoints, rather than an interest in exploring sustained behavioural change in clinical environments or patient experiences. More recent reviews have addressed specific types of pelvic examination teaching; Le Lous et al. [11] focus on simulation training and summarise impact across competence, confidence and communication. While a useful synthesis of simulation, the review does not map all approaches to teaching pelvic examination within medical education, where programmes often combine modalities.

Accordingly, there remains a need for a scoping review that moves beyond asking whether teaching approaches “work” to examine what is taught, how it is evaluated and what is missing from the way medical education defines and measures effectiveness in pelvic examination. Existing syntheses largely privilege learner confidence and competence outcomes (often short‐term), with less attention to implementation across settings with different resources and cultures, ethical and consent issues shaping learning opportunities, institutional culture and educator behaviour and inclusivity in teaching design, including communication with diverse groups. By mapping educational modalities (including combined and sequenced approaches) and synthesising how outcomes are conceptualised and measured, this review will identify gaps in evidence, highlight underexplored aspects of implementation and support curriculum leaders to design effective, culturally sensitive and feasible pelvic examination training.

Hence, we asked the following:

1. What teaching methods are reported in the literature for teaching medical students to perform pelvic examinations?
2. How are these teaching methods evaluated within the literature, and what outcomes are used to define effectiveness?
3. What barriers and enablers to implementing pelvic examination teaching methods are reported across different educational contexts?

## 2 Methods

This scoping review was conducted in accordance with the Arksey and O'Malley framework [12]. The review was prospectively registered with the Open Science Framework (OSF; DOI 10.17605/OSF.IO/2RE39) and is reported in accordance with the PRISMA‐ScR checklist (Data S1). Scoping review methodology was selected to facilitate a comprehensive, flexible approach to mapping diverse literature and because it is well‐suited to identifying research gaps.

### 2.1 Search Strategy

The search strategy was developed using the PCC (Population–Concept–Context) framework and tailored to each database with the assistance of a medical librarian. Subject headings, free‐text words, terms and synonyms relating to ‘pelvic examination’, ‘teaching’ and ‘medical students’ were included. The full search strategy is provided in Data S2. Searches were run in June 2024, and repeated in May 2026, across MEDLINE, Embase, PsycINFO (all via Ovid), ERIC (via EBSCOhost) and Scopus.

Terminology varied across studies, with terms such as professional patient, standardised patient (SP) and simulated patient used inconsistently. We use “professional patient” as an umbrella term to refer to trained individuals involved in teaching. Gynaecology Teaching Associates (GTAs) were defined as professional patients who acted as both patient and teacher, sometimes supported by faculty, whereas SPs participated primarily in simulated roles, not undergoing examination themselves and always supported by faculty.

### 2.2 Screening and Data Extraction

HS conducted the search, sourced papers and deduplicated in Endnote. Sources were imported into Rayyan and screened by two reviewers (SR/CB) in two phases (title and abstract; full text) against predetermined eligibility criteria (Table 1), with exclusion reasons documented. A random 10% sample was independently double‐screened during each phase (AH/HS), and discrepancies were resolved through wider team discussion. A Microsoft Excel data extraction form was piloted and refined before extraction.

**TABLE 1:** Inclusion and exclusion criteria.

Inclusion criteria	Exclusion criteria
‐ Published since 2000 ‐ Addresses target population of medical students/doctors ‐ Written in English ‐ Any geographical location ‐ Peer reviewed journals only ‐ Specifically addresses the practical clinical skill of either bimanual per vaginum (PV) or speculum examination for the purpose of gynaecological examination, diagnosis of pathology or screening, rather than simply learning around the anatomy	‐ Grey literature, replies/letters, magazine articles, and conference proceedings ‐ Papers written in languages other than English with no translation available ‐ Addresses the skill of vaginal examination for assessment of progression in labour

An inductive qualitative content analysis [13] was undertaken by HS, reviewed by all authors. Because studies varied substantially in how outcomes were assessed, an established educational evaluation framework was used to enable comparison. The Kirkpatrick model [14], widely used [15, 16], was applied during analysis to pragmatically categorise outcomes.

### 2.3 Reflexivity

Reflexivity was embedded throughout. The review team consisted entirely of women with direct experience of pelvic examination education and learning, including clinical educators and learners. The two undergraduate medical student authors (SR/CB) brought recent learner perspectives and first‐hand experience of pelvic examination teaching and challenges encountered in clinical practice, while senior team members (AH/HS/MB) contributed expertise as educators and researchers involved in curriculum delivery and improvement.

To support reflexive practice, reviewers engaged in regular discussion during screening and maintained reflexive notes to capture developing observations and uncertainties. Reflections were discussed within weekly team meetings, supporting consideration of how prior experiences and professional roles might shape study selection, interpretation, and categorisation. Double‐screening provided an additional mechanism to surface and challenge assumptions.

## 3 Findings

### 3.1 Screening Process

Searches returned 1541 papers in total: 1390 for the first search and 151 in the subsequent search. Screening identified 55 papers (52 originally and 3 further papers on repeat searching) which were included in the final review. The screening process is summarised in the PRISMA flowchart (Data S3).

### 3.2 Characteristics of Included Studies

The geographical focus of included studies was predominantly the Global North, with most conducted in Europe (*n* = 25) and North America (*n* = 19), alongside smaller numbers from Oceania (*n* = 5) and Asia (*n* = 4). Only two studies originated from South America, and none were identified from Africa. Fifteen were published in obstetrics and gynaecology journals and the remainder in other general journals. Nearly half of the papers (26/55, 47%) were published in education or simulation journals; participant numbers ranged from 15 to 650, including second to final‐year medical students. Included studies were published between 2002 and 2026, with publication frequency remaining broadly stable over time. The prefix ‘P’ has been added to all included references to enable cross‐checking with the full summary of studies in Data S4.

### 3.3 Overview of Findings

The teaching approaches identified by this review were grouped into two broad categories: received knowledge approaches, where learners primarily received information, and experiential approaches, where skills were practised or applied. All 55 papers described some element of experiential knowledge building, although three studies (P10, P44 and P53) adopted a comparative approach meaning received knowledge was evaluated as an isolated technique. In practice, most studies combined multiple approaches rather than evaluating single methods in isolation. Table 2 summarises teaching approaches identified across included studies.

**TABLE 2:** Summary of teaching methods identified across included studies grouped by knowledge type.

Received knowledge (*n* = 55) (35‐sources)			Experiential knowledge (*n* = 95) (55‐sources)		
Method	No.	Source	Method	No.	Sources
Lecture	22	P3; P4; P5; P6; P10; P11; P12; P14; P15; P19; P21; P23; P24; P26; P30; P36; P43; P45; P48; P49; P50; P55	Models		
			Basic	33	P1; P2; P3; P4; P6; P7; P10; P12; P13; P14; P16; P20; P21; P24; P25; P28; P30; P33; P34; P35; P36; P37; P42; P43; P44; P45; P46; P47; P48; P49; P52; P54; P55
Video	19	P1; P8; P9; P14; P19; P23; P24; P26; P33; P34; P36; P41; P42; P44; P47; P48; P49; P53; P54	Higher fidelity	8	P8; P9; P17; P18; P33; P51; P53; P55
Workbook	1	P13	Professional patients		
Directed reading	5	P1, P8, P13, P14, P23	SP	9	P5; P12; P20; P30; P32; P33; P39; P41; P46
e‐Learning	4	P1; P41; P45; P52	GTA	31	P1; P2; P3; P4; P5; P7; P8; P14; P15; P19; P21; P22; P23; P24; P25; P26; P27; P28; P29; P31; P34; P38; P40; P42; P43; P45; P46; P47; P48; P50; P54
Active demo	4	P6; P30; P43; P55	Clinical practice		
			Patients	14	P2; P11; P13; P17; P22; P25; P27; P28; P32; P33; P36; P37; P39; P54

*Note:* Many studies included more than one teaching approach within and between categories—the number of (sources) have been provided for clarity at the top of each column.

Abbreviations: GTA, Gynaecology Teaching Associates; SP, standardised patients.

*Most studies combined multiple approaches rather than evaluating single methods in isolation*.

#### 3.3.1 Received Knowledge

Two thirds (*n* = 35; 64%) of included papers described some form of received knowledge component (Table 2). Within this subset, approaches included lectures and instructional videos, which were the most frequently reported modalities (63% and 54% respectively), alongside self‐directed learning methods such as reading (14%), e‐learning (11%) and facilitated approaches including active demonstration (11%).

Five studies directed students to reading around the content areas (P1, P8, P13, P14 and P23), but only one specifically highlighted learning around the spectrum of female anatomy (P8). Overall, received knowledge approaches were commonly integrated as preparatory components within broader teaching programmes.

#### 3.3.2 Experiential Knowledge

Experiential approaches were the dominant teaching modality (Table 2), appearing in some guise in all the included papers with many including more than one approach. These were broadly categorised into model‐based learning (basic *n* = 33, 60%; high‐fidelity *n* = 8, 15%), professional patient approaches (GTAs) (*n* = 31, 56%), SPs (*n* = 9, 16%) and clinical patient encounters (*n* = 14, 25%).

*Experiential approaches were the dominant teaching modality*.

##### 3.3.2.1 Models

Model‐based teaching was described (*n* = 39‐studies, 71%) and included commercial manikins, high‐fidelity simulators, homemade models and models combined with adjuncts such as virtual reality or ultrasound. Six studies described model‐based teaching as a standalone approach, two as part of singular method studies (P16 and P51) and four as part of comparative studies (P35, P44, P53 and P17); most combined models with additional teaching methods. Basic commercial manikins were frequently used, while some studies described student‐made low‐cost models to improve anatomical realism (P16), including 3D‐printed models used as both simulation tools and a pedagogical exercise in model construction (P35). Higher fidelity approaches incorporated virtual reality or technological enhancements, for example, pressure sensors or visualisation components (P51, P17, P9, P8, P53 and P55). Hybrid approaches were also reported, combining models with SPs positioned behind the manikin to integrate communication with technical practice (P20, P46, P30 and P12). Overall, models were widely used as part of mixed teaching strategies.

##### 3.3.2.2 Professional Patients

Professional patient teaching approaches were also commonly reported (*n* = 38 studies, 69%). Terminology varied across studies; in this review, professional patients included SPs and GTAs. GTA‐led teaching was frequently described as positively received by students, with reported outcomes including increased confidence, reduced anxiety, and perceived psychological safety (P1, P45, P38, P29, P31 and P24). Some studies identified practical challenges, including recruitment, funding, and sustainability (P2, P39, P44 and P48).

Comparative studies reported similar levels of objectively assessed competence between students trained with GTAs and SPs in some contexts (P5), while others reported differences in student perceptions or performance that did not consistently persist over time (P21 and P27). GTA teaching was evaluated positively as a singular method in three studies (P29, P31 and P38). In sum, professional patient approaches were used across diverse educational contexts and were often integrated with other teaching modalities.

##### 3.3.2.3 Clinical Practice

Clinical patient encounters were reported in 14 studies (25%) (Table 2). These included routine clinical placements, dedicated student clinics and teaching conducted on anaesthetised patients. Only one study (P37) examined clinical practice as a standalone approach. Several studies emphasised the authenticity of clinical encounters and opportunities for repeated practice (P32, P22 and P28). Dedicated student clinics were associated with higher perceived teaching quality despite similar numbers of examinations performed (P11).

Cross‐national studies described variation in practice, including differing use of professional patients and teaching on anaesthetised patients across settings (P33, P39, P2 and P14). Historical analyses suggested a shift over time toward greater use of professional patients and GTA‐led teaching models. Overall, clinical practice was described as an important component of learning but was rarely evaluated independently.

#### 3.3.3 Combined and Sequenced Approaches

As shown in Table 3, most studies (44/55, 80%) combined multiple teaching approaches either sequentially or concurrently. Typical sequences included initial received knowledge‐based preparation (e.g., lectures or videos), followed by experiential model‐based practice and subsequent professional patient or clinical encounters. Combined approaches were often described as allowing progressive development of technical skills alongside communication and patient‐centred competencies.

**TABLE 3:** Summary of teaching approaches identified across included studies.

Combined (*n* = 34)			Comparative (*n* = 16)		
Approach	*n*	Source(s)	Approach	*n*	Source(s)
RK + M	4	P6; P9; P49; P52	RK^a^ vs. RK + M	1	P10
RK + SP	1	P41	M^a^ vs. CP^a^	1	P17
RK + GTA	4	P15; P19; P23; P26	SP^a^ vs. CP^a^	1	P32
RK + CP	1	P11	CP^a^ vs. CP + M	1	P37
RK + M + GTA	7	P8; P24; P34; P42; P43; P45; P48	RK^a^ vs. M^a^	2	P44; P53
RK + M + CP	2	P13; P36	M (Com)^a^ vs. M(3D)^a^	1	P35
RK + GTA + M + CP	1	P54	GTA^a^ vs. SP^a^	1	P5
RK + GTA + USS	1	P50	GTA^a^ vs. M + CP	1	P28
M + debrief	1	P18	GTA^a^ vs. M + SP	1	P46
M + SP	1	P20	RK + M vs. RK + GTA	2	P3; P4
M + GTA + CP + R	1	P25	RK + M + SP(q) vs. RK + M + SP(l)	1	P12
GTA + CP	2	P22; P27	RK + M vs. RK + M + SP	1	P30
Other^b^	8	P1; P2; P7; P14; P33; P39; P40; P47	RK + M vs. RK + M + GTA	1	P21

**Single (*n* = 5)**			RK + M vs. RK + M (VR)	1	P55
**Approach**	***n***	**Source**			
GTA	3	P29; P31; P38			
Models	2	P16; P51			

Abbreviations: CP, clinical patients; GTA, gynaecology teaching associates; M, model; M(3D), 3D printed model; M (Com), commercial model; R, reflection; RK, received knowledge; SP, standardised patient; SP(l), standardised patient (loud); SP(q), standardised patient (quiet); USS, ultrasound; VR, virtual reality.

^a^ Denotes a single method used within a comparative analysis.

^b^ Category includes literature and curriculum reviews where the method of combination in practice was unclear.

#### 3.3.4 Reflective and Debriefing Approaches

Several studies explicitly described directed feedback, reflective discussion or debriefing as components of teaching (P14, P15, P18, P25 and P54), although these were rarely evaluated as standalone interventions. Reflection typically occurred following simulation or professional patient encounters and was used to support discussion of technical performance, communication and sensitive topics such as sexuality, consent or cultural considerations (P25).

Group discussion and facilitated debriefing were often embedded within broader teaching programmes rather than framed as distinct pedagogical strategies (P14, P15 and P25). Where described, reflective elements were positioned as supporting integration of technical and relational aspects of pelvic examination learning, particularly following experiential activities. Despite frequent inclusion within combined teaching approaches, few studies evaluated the independent impact of reflection, feedback or debriefing on learner outcomes.

#### 3.3.5 Evaluation of Teaching Approaches

Studies used a wide range of outcome measures to evaluate teaching. Outcomes most commonly focused on self‐reported learner‐centred measures, including confidence, anxiety and perceived competence, with fewer studies (*n* = 17) employing objective skills assessment (P4, P5, P10, P12, P17, P18, P21, P25, P28, P30, P37, P41, P43, P44, P45, P46 and P50) and only one specially referencing the engagement of GTAs in the assessment process (P46). For comparison, studies were mapped to Kirkpatrick levels. Most studies (*n* = 38, 69%) evaluated outcomes at Level 2 (learning), while seven (13%) focused on learner reaction or satisfaction (level 1). Seven studies (13%) lacked sufficient detail for classification, and three (5%) had no evaluative element. No studies examined behavioural change in clinical practice (Level 3) or demonstrated direct patient outcome effects (Level 4).

#### 3.3.6 Barriers and Enablers to Implementation of Teaching Approaches

Several studies described factors that influenced the implementation and delivery of pelvic examination teaching. These barriers and enablers were related to educational setting, resource availability, organisational culture and the structure of clinical learning environments.

Structured teaching environments were described as facilitating implementation and learner engagement. Dedicated student clinics were associated with higher perceived teaching quality, even when students performed similar numbers of examinations to those in routine placements (P11). Clinical encounters were described as valuable learning opportunities, particularly where students were supported to practise repeatedly and gain familiarity with normal anatomy (P32, P22 and P28).

Professional patient programmes, including GTA‐led teaching, were frequently reported as enabling psychologically safe learning environments in which students felt more comfortable asking questions, receiving feedback and practising sensitive skills (P42, P45, P38, P29, P31 and P24). Several studies also suggested that prior exposure to structured teaching approaches, such as models or GTAs, increased students' willingness to engage in further practice during clinical placements (P45 and P24).

Sequential or combined teaching approaches were described as facilitating implementation by supporting progression from preparatory knowledge‐based learning to simulation and subsequently clinical practice (P8 and P47). Resource‐related constraints were commonly reported barriers to implementation. Studies described challenges relating to recruitment, training and sustainability of GTA programmes (P48). Where professional patient approaches required faculty supervision, increased staffing demands and loss of clinical revenue were identified as additional challenges (P5).

Clinical environments also presented barriers. One study described efforts to minimise visible student learning during clinical encounters to preserve perceived clinician expertise, which resulted in reduced supervision and limited feedback opportunities (P48). Variability in clinical exposure was reported across settings, with some students having limited opportunities to perform pelvic examinations during placements (P37 and P54).

Organisational and cultural context influenced implementation across countries. Survey studies demonstrated variation in the extent to which medical schools relied on professional patients, manikin‐based teaching, or clinical practice (P33, P39, P2 and P14). Differences were also reported in practices surrounding teaching pelvic examination on anaesthetised patients; while this was explicitly described in United Kingdom (UK) data (P2), other studies highlighted a growing shift away from educational examination under anaesthesia (EAU), reflecting changing ethical expectations around consent but also raising concerns about reduced opportunities for skills acquisition (P54).

## 4 Implications

This scoping review maps the range of teaching approaches to pelvic examination within medical education, examining how effectiveness is conceptualised and evaluated and identifying implementation factors influencing delivery. Here, we also consider the implications of these findings on equity. While not an original focus of our review, the cultural dimensions of teaching design emerged as of significant importance, and we wish to draw attention to the implications of these findings to promote further work. The three categories of implications we consider here are interconnected: implementation choices shape what can be evaluated, and both are influenced by the cultural and institutional contexts in which training is delivered.

### 4.1 Cost and Practicality

This review identified a wide range of teaching approaches, with most studies combining multiple methods rather than relying on single interventions. However, implementing these approaches is shaped by practical considerations, particularly cost and resource availability. Teaching pelvic examinations can be resource‐intensive, particularly when using GTAs, who act as both teacher and patient [17, 18]. While GTAs offer high‐quality experiential training, cost may limit accessibility [19, 20]. Low‐cost interventions, such as homemade models, may provide comparable learner outcomes, though these require further validation [21]. Teaching clinics, though well‐received by students, reduce patient throughput, which represents a challenge in overstretched healthcare systems. Additionally, faculty time diverted from clinical care to teaching exacerbates existing workforce pressures [22]. Cost‐effectiveness analyses are complicated by the difficulty of tracking long‐term impacts on patient outcomes [23]. For instance, cervical cancer screening saves 2000 lives annually in the UK [24], highlighting the potential downstream importance of competent examination skills. However, without skill retention, the value of these teaching investments diminishes [25].

*Cost‐effectiveness analyses are complicated by the difficulty of tracking long‐term impacts on patient outcomes*.

### 4.2 Defining and Evaluating Effectiveness

Most included studies evaluated learner‐centred outcomes such as confidence, anxiety or perceived competence. Mapping outcomes to Kirkpatrick levels demonstrated a predominance of Levels 1 and 2 outcomes, with limited attention to behavioural change or patient‐level impact. This pattern reflects wider trends in medical education research. Barrett et al. [26], in a review of workplace‐based assessment, similarly found that only a small proportion of outcomes were measured at higher Kirkpatrick levels (3 or 4), with most focusing on learning outcomes. This is likely as higher level outcomes, such as impact on patient care, can be challenging to measure and may only become apparent over extended timescales [23]. As a result, lower level outcomes remain more common, even though these do not necessarily translate into sustained behavioural change.

These findings, therefore, raise important questions about how effectiveness should be defined in pelvic examination education. Based on this review, we propose that meaningful evaluation should move beyond immediate learner confidence to encompass the following: (1) objective assessment of technical competence at the point of clinical practice, rather than only at the end of a teaching session; (2) longitudinal follow‐up of whether skills are retained and applied; and (3) patient‐reported outcomes, including whether patients feel examinations are conducted respectfully, with adequate consent and with appropriate sensitivity. Pragmatically, proxy measures (e.g., rates of clinically indicated examinations being performed, cervical screening uptake and patient satisfaction with pelvic examinations) may offer tractable intermediate endpoints. We appreciate that these are not straightforward to measure, but without aiming for higher level outcomes within evaluation, we risk revising our approach to education based on data relating only to learner satisfaction, rather than patient outcomes. In the context of intimate examinations like pelvic examination, patient experiences are central to competent practice; a technically correct examination that is distressing or inadequately explained can cause more harm than good long‐term.

### 4.3 Equity, Inclusion and Cultural Context

Beyond practical considerations, this review highlights how pelvic examination teaching is shaped by broader cultural and equity‐related factors. While teaching methods were often evaluated in terms of learner confidence or technical competence, fewer studies considered how training environments reflect diverse patient identities and experiences.

Pelvic examination training must evolve to reflect diverse patient populations. Models remain predominantly white [27], although some efforts to introduce varied skin tones are emerging. Similarly, few studies explicitly addressed the spectrum of normal female anatomy, potentially limiting students' understanding of anatomical diversity; an issue was recently reported by practicing clinicians [28]. Although a shift toward inclusivity is evident in some settings [29], cisnormative assumptions remain embedded within much literature. For example, relatively few studies considered how teaching could better prepare students to care for transgender men or gender‐diverse patients. Hybrid approaches, combining SPs alongside manikins, may offer opportunities to integrate communication and history‐taking with anatomically appropriate examination practice while supporting discussions around inclusivity.

*Pelvic examination training must evolve to reflect diverse patient populations*.

Cultural context also influences what teaching approaches are feasible or acceptable, particularly within increasingly diverse and internationalised medical education environments. As medical schools recruit students from a wide range of cultural backgrounds, learners may enter training with differing expectations of patient autonomy, consent, hierarchy and acceptable levels of patient involvement in teaching. Students trained in one cultural context but learning medicine in another may experience uncertainty or tension when local norms around patient engagement differ from their prior assumptions. In some settings, GTA‐based teaching may be stigmatised, requiring adaptation of teaching models to avoid harm to participants [30, 31]. Broader cultural attitudes toward consent and clinical learning further shape practice. In certain contexts, active learning during clinical encounters may be minimised to preserve perceptions of clinician expertise or reduce perceived patient discomfort, limiting opportunities for open supervision and feedback [31]. This highlights the need for culturally responsive teaching models that explicitly address differing expectations and support students in navigating these complexities rather than assuming shared understandings of professionalism and patient partnership.

Historical approaches to pelvic examination teaching also reflect wider shifts in culture around consent and autonomy. EUA for teaching purposes has traditionally been used as a way to facilitate learning without causing patient discomfort, and arguments both for and against this practice in pelvic examination teaching continue to be debated in the literature [32]. However, increasing scrutiny has highlighted that the absence of discomfort does not remove the ethical requirement for explicit informed consent. Discussions across multiple settings have emphasised that trust, transparency and respect for patient autonomy must underpin any examination performed under general anaesthesia [33, 34, 35], with patients expressing a clear preference to remain awake and involved regardless of discomfort [36].

Despite this, surveys suggest that pelvic EUA for teaching purposes may still occur without clear consent, even where medical students themselves recognise the practice as unethical [37]. Internationally, this has prompted growing calls for legislative and institutional change, including debate in Australia about whether explicit consent for educational pelvic EUA should be legally mandated, following moves already seen in parts of the United States [38]. This changing discourse reflects a broader cultural shift: Practices that were historically normalised within hidden curricula are increasingly being challenged as incompatible with contemporary expectations of respectful care.

### 4.4 Directions for Future Research

We identified several literature gaps. Effective feedback is recognised as essential for developing pelvic examination skills, and approaches such as sensor‐equipped models, faculty‐guided teaching and GTA‐led sessions may help learners refine technique. However, pelvic examination presents unique challenges because much of the examination is not directly visible to either learner or educator. As a result, students may reproduce the physical steps demonstrated to them without fully understanding what they are expected to feel, interpret or identify clinically. This raises questions about how teaching approaches support not only technical performance but also the development of clinical reasoning and recognition of abnormal findings.

Although reflection and debriefing were included within some teaching programmes, their independent contribution to learning outcomes was rarely evaluated. Further research examining reflective and feedback processes may help clarify how learners integrate technical and relational aspects of examination practice.

High‐cost teaching approaches, such as virtual reality and sensor‐enhanced models, offer exciting potential but cannot fully replicate human interaction. Simulation plays an important role; however, clinical encounters remain central given the prevalence of pelvic examination in everyday practice.

Patient perspectives were largely absent from the literature. While some studies suggested patients valued respectful communication and student involvement, patient input and voice—particularly across different cultural contexts—remain underrepresented [39]. This restricts our understanding of how educational practices align with patient expectations/experiences and represents an important gap.

### 4.5 Wider Implications for Health Professions Education

While this review focuses on undergraduate medical education, its findings have relevance for a broader range of health professionals who perform pelvic examinations, including nurses, midwives and nurse practitioners. Many of the challenges identified within this review are not unique to medicine, for example, resource constraints, the ethical complexity of learning intimate examination skills. Interprofessional approaches to pelvic examination training, where students from different professions learn together, remain largely unexplored but may offer practical and pedagogical advantages, including shared use of resources such as GTAs, and opportunities to model collaborative, patient‐centred care.

For health professions clinical teachers, there are several practical considerations. First, no single teaching method is superior; combined and sequenced approaches that progress from received knowledge through simulation to supervised clinical practice appear most consistently described. Structured, psychologically safe settings are critical to learning and facilitate engagement from learners, including dedicated teaching clinics and GTA‐led sessions where resources allow. Curricula developers should consider inclusivity within the context of pelvic examination skills; diverse anatomical models, explicit teaching around consent and communication for diverse patient groups and consideration of the cultural contexts in which students are learning can help promote an inclusive approach to practice amongst learners. Finally, evaluation should extend beyond immediate learner confidence. Clinical teachers, and institutions, should reflect carefully on how to assess the retention of pelvic examination skills, and their application in practice, including the impact on patient experiences [40].

### 4.6 Limitations

In line with Arksey and O'Malley [12], formal quality appraisal was not undertaken. This is consistent with scoping review methodology which prioritises mapping breadth of evidence over critical evaluation. However, as a result, the robustness of individual studies cannot be determined; areas of apparent evidence density may reflect weaker studies, and the absence of a gap does not necessarily indicate a methodologically robust literature. Included studies were heterogeneous in design, terminology and outcome measures, which limited direct comparison across interventions. Terminology describing professional patients varied substantially between studies, requiring pragmatic categorisation. The review was limited to English‐language publications, which may have introduced language bias. In addition, many studies relied on self‐reported outcomes, and relatively few incorporated objective assessments or long‐term follow‐up, restricting conclusions about sustained behavioural change or impact on patient outcomes.

## 5 Conclusion

Pelvic examinations are essential, potentially life‐saving procedures that must be taught effectively. This scoping review found that undergraduate medical education commonly combines didactic teaching, experiential learning and clinical practice, balancing technical skill development with communication, consent and patient comfort. However, evaluation remains largely focused on learner outcomes, with limited evidence of effects on behaviour or patient care. Future educational approaches should be feasible, cost‐effective, accessible, culturally sensitive and inclusive of diverse patient populations, while being tailored to local contexts and supported by ongoing evaluation. Rather than identifying a single “best” teaching method, the priority should be understanding how educational interventions are implemented, sustained and aligned with the realities of clinical practice.

*Pelvic examinations are essential, potentially life‐saving procedures that must be taught effectively*.

## Author Contributions

**Carol Boutrous:** formal analysis, writing – original draft, writing – review and editing. **Shweta Rao:** formal analysis, writing – original draft, writing – review and editing. **Anna Harvey Bluemel:** conceptualization, supervision, writing – review and editing, writing – original draft, formal analysis. **Megan E. L. Brown:** writing – review and editing, writing – original draft. **Heidi Stelling:** conceptualization, formal analysis, funding acquisition, project administration, writing – original draft, writing – review and editing, supervision.

## Funding

No specific funding was received for this project; however, the involvement of undergraduates (CB and SR) in the project was funded by a Newcastle University Student Employment on Campus (SEOC) internship scheme.

## Ethics Statement

The authors have nothing to report.

## Consent

The authors have nothing to report.

## Conflicts of Interest

The authors declare no conflicts of interest.

## Supporting information


**Data S1:** PRISMA Reporting Checklist.

**Data S2:** Search Strategy.

**Data S3:** PRISMA Flow Diagram.

**Data S4:** Summary of Included Sources.

## Data Availability

The data that support the findings of this study are available from the corresponding author upon reasonable request.
