# Supplementary material for: Pelvic Examination in Undergraduate Medical Education: A Scoping Review
Source: Clin Teach. 2026 Jul 8;23(4):e70475. doi: 10.1111/tct.70475 (PMC13343211; doi:10.1111/tct.70475)
Supplement: Supplementary file 2 — Data S2: Search Strategy. [file TCT-23-e70475-s001.docx]

Supplementary File 2: Search Strategy

# Medline

| **Ovid MEDLINE(R) and In-Process, In-Data-Review & Other Non-Indexed Citations 1946 to June 16, 2024**  **Accessed via ovid.com \| Run Date: 17^th^ June 2024** | |
| --- | --- |
| **Line#** | **Search String** |
| 1 | Student, Medical/ or (medical undergrad* or student doctor*).ti,ab,kf. |
| 2 | Gynecology/ed or Gynecological examination/ or exp Genitalia, Female/ or exp pelvis/ or  (vaginal speculum or per vagin* or bimanual or ((pelvic or intimate or vaginal or genital) adj3 exam*)).ti,ab,kf. |
| 3 | education/ or curriculum/ or health education/ or sex education/ or education, medical/ or education, medical, undergraduate/ or teaching rounds/ or clinical competence/ or schools, medical/ or exp teaching/ or exp simulation training/ or learning/ or models, theoretical/ or models, educational/ or (course content or curriculum development* or education* program* or education* theor* or learning environment*). ti,ab,kf, |
| 4 | 1 AND 2 AND 3 |

**Titles: 361**

# Embase

| **EMBASE 1974 – 2024 June 14^th^**  **Accessed via ovid.com \| Run Date: 17^th^ June 2024** | |
| --- | --- |
| **Line#** | **Search String** |
| 1 | medical student/ or (medical undergrad* or student doctor*).ti,ab,kw. |
| 2 | gynecological examination/ or pelvic examination/ or exp female genital system/ or pelvis/ or vaginal speculum/ or (per vagin* or bimanual or ((intimate or vaginal or genital) adj3 exam*)).ti,ab,kw. |
| 3 | education/ or course content/ or curriculum/ or curriculum development/ or education program/ or educational theory/ or health education/ or learning environment/ or medical education/ or medical school/ or teaching round/ or exp clinical education/ or "outcome of education"/ or sexual education/ or exp simulation training/ or exp teaching/ or clinical competence/ or learning/ or educational model/ |
| 4 | 1 AND 2 AND 3 |

**Titles: 408**

# Psych Info

| **APA PsychInfo 1806 to June Week 1 2024**  **Accessed via ovid.com \| Run Date: 17^th^ June 2024** | |
| --- | --- |
| **Line#** | **Search String** |
| 1 | medical students/ or (medical undergrad* or student doctor*).ti,ab,id. |
| 2 | physical examination/ or exp female genitalia/ or (pelvis or per vagin* or bimanual or vaginal speculum or ((gyn*ecology* or pelvic or intimate or vaginal or genital) adj3 exam*)).ti,ab,id. |
| 3 | medical education/ or education/ or curriculum/ or health education/ or sex education/ or exp teaching/ or exp competence/ or simulation/ or learning/ or “theories of education”/ or (course content or curriculum development* or education* program* or learning environment* or medical school).ti,ab,id. |
| 4 | 1 AND 2 AND 3 |

**Titles: 114**

# Scopus

| **Scopus**  **Accessed via scopus.com \| Run Date: 17^th^ June 2024** | |
| --- | --- |
| **Line#** | **Search String** |
| Topic 1 | TITLE-ABS-KEY(“medical student*” OR “medical undergraduate*” OR “student doctor”) |
| AND  Topic 2 | TITLE-ABS-KEY (“gyn*ecological examination” OR “pelvic exam*” OR “female genital system” OR “pelvis” OR “vaginal speculum” OR “intimate exam*” OR “vaginal exam*” OR “per vagin*” OR “genital exam*” OR bimanual) |
| AND  Topic 3 | TITLE-ABS-KEY (education OR “course content” OR curriculum OR “curriculum development” OR “education program” OR “educational theory” OR “health education” OR “learning environment” OR “medical education” OR “medical school” OR “teaching round” OR “clinical education” OR "outcome of education" OR “sexual education” OR “simulation training” OR “teaching” OR “clinical competence” OR “learning” OR “educational model”) |

**Titles: 487**

# ERIC

| **ERIC (Education Resource Information Center)**  **Accessed via EBSCOHost.com \| Run Date: 17^th^ June 2024** | |
| --- | --- |
| **Line#** | **Search String** |
| Topic 1 | TX (“medical student*” OR “medical undergraduate*” OR “student doctor”) |
| AND  Topic 2 | TX (“gyn*ecological examination” OR “pelvic exam*” OR “female genital system” OR “pelvis” OR “vaginal speculum” OR “intimate exam*” OR “vaginal exam*” OR “per vagin*” OR “genital exam*” OR bimanual) |
| AND  Topic 3 | TX (education OR “course content” OR curriculum OR “curriculum development” OR “education program” OR “educational theory” OR “health education” OR “learning environment” OR “medical education” OR “medical school” OR “teaching round” OR “clinical education” OR "outcome of education" OR “sexual education” OR “simulation training” OR “teaching” OR “clinical competence” OR “learning” OR “educational model”) |

**Title: 19**

# Search Update

No changes to the strategy were made but the search was rerun on **5^th^ May 2026** in each database to look for new titles prior to publication - results are tabulated below.

| **Database** | **Additional Title** |
| --- | --- |
| Medline | 55 |
| Embase | 50 |
| Psych Info | 2 |
| Scopus | 42 |
| ERIC | 2 |
| **Total** | **151** |
