# Supplementary material for: Pelvic Examination in Undergraduate Medical Education: A Scoping Review
Source: Clin Teach. 2026 Jul 8;23(4):e70475. doi: 10.1111/tct.70475 (PMC13343211; doi:10.1111/tct.70475)
Supplement: Supplementary file 3 — Data S3: PRISMA Flow Diagram. [file TCT-23-e70475-s002.docx]

**Identification of studies via databases and registers**

Records identified from:

**Databases (Total = 1541) [1390+151]**

Medline (n = 417) [362+55]

Embase (n = 458) [408+50]

PsycInfo (n = 116) [114+2]

Scopus (n = 529) [487+42]

ERIC (n = 21) [19+2]

Records removed *before screening*:

Duplicate records removed

(n = 425) [385+40]

**Identification**

Records excluded:

Automated exclusion* (n = 130) [130+0]

Manual exclusion (n = 858) [759+99]

*Date <2000

Records screened

(n = 1116) [1005+111]

Reports not retrieved

(n =2) [0+2]

Reports sought for retrieval

(n = 128) [116+12]

**Screening**

Reports excluded:

Publication Type (n = 10) [7+3]

Population* (n=5) [5+0]

Insufficient Data (n=1) [1+0]

Language (n = 5) [5+0]

Content** (n =50) [46+4]

*Includes papers where population was mixed and not analysed separately

** Includes papers where the pelvic examination data could not be separated from another intimate examination

Reports assessed for eligibility

(n = 126) [116+10]

Reports of included studies

(n = 55) [52+3]

**Included**

**[Square bracket indicated the number from the first search in June 2024 and repeat search in May 2026 respectively.]**

Source: Page MJ, et al. BMJ 2021;372:n71. doi: 10.1136/bmj.n71.

This work is licensed under CC BY 4.0. To view a copy of this license, visit <https://creativecommons.org/licenses/by/4.0/>
