# Supplementary material for: Pelvic Examination in Undergraduate Medical Education: A Scoping Review
Source: Clin Teach. 2026 Jul 8;23(4):e70475. doi: 10.1111/tct.70475 (PMC13343211; doi:10.1111/tct.70475)
Supplement: Supplementary file 4 — Data S4: Summary of Included Sources. [file TCT-23-e70475-s004.docx]

Supplementary File 4 – Summary of Included Sources

**Abbreviations** - GTA: Gynaecology Teaching Associate, SP: Standardised Patient, GA: General Anaesthetic, OPD: Out-Patient Department, Adj: Adjunct, VR: Virtual Reality.

| **DEMOGAPHICS** | | | | **PELVIC TEACHING METHODS** | | | | | | **EVALUATION** | |
| --- | --- | --- | --- | --- | --- | --- | --- | --- | --- | --- | --- |
|  |  |  |  |  | **Experiential Knowledge** | | |  |  |  |  |
| **No.** | **Author** | **Title** | **Year** | **Received Knowledge (RK)** | **Model (M)** | **Professional Patient (PP)** | **Clinical Practice (CP)** | **Other** | **Approach** | **Evaluation Method** | **Kirkpatrick Level** |
| P1 | Abirami Kirubarajan, Xinglin Li, Tiffany Got, Matthew Yau, and Mara Sobel | Improving Medical Student Comfort and Competence in Performing Gynecological Exams: A Systematic Review | 2021 | Reading  eLearning  Videos | Model | GTA | - | - | Literature review | NA | NA |
| P2 | Aisha Janjua, Paul Smith & T. Justin Clark | A cross-sectional study on teaching pelvic examination in medical schools in the UK (the COTES study) | 2018 | - | Model | GTA | CP (OPD and Theatre ↓GA) |  | Review of UK Curriculums via cross-sectional survey | Self-assessment using online survey | Insufficient Data |
| P3 | Aisha Janjua, Tracy Roberts, Nicola Okeahialam, T Justin Clark | Cost-effective analysis of teaching pelvic examination skills using Gynaecology Teaching Associates (GTAs) compared with manikin models (The CEAT Study) | 2018 | Lecture | Model | GTA | - | - | Comparative: RK+M vs RK+GTA | Self-assessment of confidence & competence using pre- and post-training questionnaires | 2 |
| P4 | Aisha Janjuaa , P. Smithb , J. Chub , N. Rautc , S. Malickd , I. Gallosb , R. Singhb , S. Irania , J.K. Guptab, J. Parlee , T.J. Clarkb, | The effectiveness of gynaecology teaching associates in teaching pelvic examination to medical students: a randomised controlled trial | 2016 | Lecture | Model | GTA | - | - | Comparative: All RK + either M or GTA | Formal assessment using checklist, self-assessment of confidence using visual analogue scale | 2 |
| P5 | Archana Pradhan, Gary Ebert, Pamela Brug, David Swee & Cande V. Ananth | Evaluating pelvic examination training: Does faculty involvement make a difference? a randomized controlled trial | 2010 | Lecture | - | GTA SP | - | - | Comparative: GTA vs SP + faculty | 3 station OSCE with checklist | 2 |
| P6 | Arunaz Kumar, Carole Gilmour, Debra Nestel, Robyn Aldridge, Gayle McLelland and Euan Wallace | Can we teach core clinical obstetrics and gynaecology skills using low fidelity simulation in an interprofessional setting? | 2014 | Lecture  Active Demo | Model | - | - | - | Combined: RK + M | Self-assessment to rate student experience | 1 |
| P7 | Arunaz Kumar, Carole Gilmour, Debra Nestel, Robyn Aldridge, Gayle McLelland and Euan Wallace | Simulation training for pelvic examination: A systematic review | 2023 | - | Model | GTA | - | - | Literature review | NA | NA |
| P8 | Carla M. Pugh, Eniola T. Obadina & Kofi A. Aidoo | Fear of causing harm: Use of mannequin-based simulation to decrease student anxiety prior to interacting with female teaching associates | 2009 | Reading  Video | Model e-Pelvis | GTA | - | - | Combined: RK + M + GTA | Self-assessment using surveys; anxiety | 2 |
| P9 | Carla M. Pugh, Patricia Youngblood | Development and validation of assessment measures for a newly developed physical examination simulator | 2002 | Video | e-Pelvis | - | - | - | Combined: RK + M | Formal assessment - mean accuracy score, reliability score, written exam paper | Insufficient Data |
| P10 | Galo Sánchez del Hierro, Roy Remmen, Veronique Verhoeven, Paul Van Royen, Kristin Hendrickx | An improvement in medical undergraduate education in Ecuador throught hands-on training in perinatal skills | 2017 | Lecture | Model | - | - | - | Comparative: RK +/- M | OSCE stations final station | 2 |
| P11 | Gary Sutkin and Kenneth Dzialowski | A gynaecologic clinic dedicated to student teaching | 2013 | Lecture | - | - | CP | - | Combined: RK + CP (one-to-one vs group) | Self-assessed; asked to rate overall learning experience | 2 |
| P12 | Glenn D Posner, Stanley J Hamstra | Too much small talk? Medical students' pelvic examination skills falter with pleasant patients | 2013 | Lecture | Model | SP | - | - | Comparative: All RK + M vs M/quiet SP vs M/engaged SP | Formal assessment - technical and communication skills rated by 8 professionals | 2 |
| P13 | Helen Paterson, Kristin Kenrick and Don Wilson | Teaching the Y generation obstetrics and gynaecology skills: a survey of medical students' thoughts on a new program | 2012 | Reading Workbook | Model | - | CP | - | Combined:  RK + M +CP | Self-assessment pre- and post-training surveys; competence, confidence, interest in O&G, feedback on teaching materials | 2 |
| P14 | Hiske Van Ravesteijn, Emer Hageraats & Jan-Joost Rethans | Training of the gynaecological examination in the Netherlands | 2009 | Video  Lecture Reading | Model | GTA | - | Group discussion | Curriculum review in Netherlands | Self-assessed using questionnaires and discussions | 2 |
| P15 | Ion E. Kamemoto, Kathleen Kane and LeighAnn C. Frattarelli | Pelvic examination teaching: linking medical student professionalism and clinical competence | 2003 | Lecture | - | GTA | - | Group discussion | Combined: RK + GTA | Self-assessment using discussion & written feedback from students | Insufficient Data |
| P16 | James E Benson, Allix M Hillebrand, Tamika Auguste | Design and evaluation of a low-cost speculum examination training model | 2017 | - | Model - Homemade | - | - | - | Singular | Self-assessed; usefulness of model | 2 |
| P17 | Janjua, Smith, Chu, Raut, Malick, Gallos, Singh, Irani, Gupta, Parle, Clark | Bimanual vaginal examination: Using innovation through cognitive task analysis to standardise practise and enhance teaching | 2016 | - | Model with internal video and a semi-transparent vaginal cavity | - | CP | - | Comparative: M vs CP | Formal assessment of student performance | 2 |
| P18 | Jennifer Pearson, Amy Greminger, Emily Onello, Sandy Stover | Teaching the Evaluation of Female Pelvic Pain: A Hands-On Simulation to Reinforce Exam Skills and Introduce Transvaginal Ultrasound | 2021 | - | Pelvic Task Traier Sim Mom + USS | - | - | Debrief | Combined: M + Debief | Formal assessment of pelvic examination simulation with faculty critical action checklist, a student survey, and multiple-choice examination questions | 2 |
| P19 | Jenny Gleisner, Karin Siwe | Differences in teaching female and male intimate examinations: A qualitative study | 2020 | Lecture  Video | - | GTA | - | - | Combined: RK + GTA | Self-assessment using interviews; student experience | 2 |
| P20 | Jenny Higham, Debra Nestel, Martin Lupton and Roger Kneebone | Teaching and learning gynaecology examination with hybrid simulation | 2007 | - | Model | SP | - | - | Combined: M + SP | Self-assessment of competence | 2 |
| P21 | JMN Duffy, S Chequer, A Braddy, S Mylan, A Royuela, J Zamora, J Ip,f S Hayden, M Showell, P Kinnersley, R Chenoy, OM Westwood, KS Khan, A Cushingg | Educational effectiveness of gynaecological teaching associates. A multi-centre randomised controlled trial | 2016 | Lecture | Model | GTA | - | - | Comparative:  RK+M +/- GTA | Summative OSCE (assessing technical and interpersonal skills); self-assessed comfort and confidence | 2 |
| P22 | Johanna Danielsson, Cecilia Hadding, Martin Fahlström, Ulrika Ottander, David Lindquist | Medical students' experiences in learning to perform pelvic examinations: a mixed-methods study | 2021 | - | - | GTA | CP | - | Combined: GTA + CP | Group discussions | 1 |
| P23 | Karin Siwe, Barbro Wijma , Charlotte Sile, Carina Bertero | Performing the first pelvic examination: Female medical students' transition to examiners | 2007 | Reading Lecture  Video | - | GTA | - | - | Combined: RK + GTA | Interviews to explore students' satisfaction | 2 |
| P24 | Karin Siwe, Klaas Wijma, Martin Stjernquist, Barbro Wijma | Medical students learning the pelvic examination: Comparison of outcome in terms of skills between a professional patient and a clinical patient model | 2007 | Lecture  Video | Model | GTA | - | - | Combined: RK + M + PP | Self-assessed; rating distress levels (Gynaecologic Examination Distress Questionnaire [GyExDQ]) and adequacy of teachers | 2 |
| P25 | Kjell Wånggren, Aino Fianu Jonassen, Sonja Andersson, Gunilla Pettersson, Kristina Gemzell-Danielsson | Teaching pelvic examination technique using professional patients: A controlled study evaluating students skills | 2010 | - | Model | GTA | CP | Reflection | Combined: M + GTA +CP + Reflection | Practical test for technical and communication skills | 2 |
| P26 | Kjell Wånggren, Gunilla Pettersson, György Csemiczky & Kristina Gemzell-Danielsson | Teaching medical students gynaecological examination using professional patients - Evaluation of students' skills and feelings | 2005 | Lecture  Video | - | GTA | - | - | Combined: RK + GTA | Self-assessed; questionnaires for students, teachers, and professional patients; technical skills, communication skills, attitude | 2 |
| P27 | Kjell Wånggren, Gunilla Pettersson, Kristina Gemzell-Danielsson | Medical students learning the pelvic examination: Evaluation of a clinical patient model | 2010 | - | - | GTA | CP | - | Combined: GTA+ CP | Self-assessed; confidence, technical skills, emotional states | 2 |
| P28 | Kristin Hendrickx, Benedicte De Winter, Wiebren Tjalma, Dirk Avonts, Griet Peeraer & Jean-Jacques Wyndaele | Learning intimate examinations with simulated patients: the evaluation of medical students' performance | 2009 | - | Models | GTA | CP | - | Comparison: GTA vs M and CP | Formal OSCE assessment using detailed checklist, self-assessment of students using questionnaire on competence and experience, feedback from GTAs | 2 |
| P29 | Kristin Hendrickx, Benedicte Y. De Winter, Jean-Jacques Wyndaele, Wiebren A.A. Tjalma, Luc Debaene, Bert Selleslags, Frieda Mast, Philippe Buytaert, Leo Bossaert | Intimate examination teaching with volunteers: implementation and assessment at the University of Antwerp | 2006 | - | - | GTA | - | - | Singular | Self-assessment using questionnaire | 2 |
| P30 | Kristyn Manley, Sian Edwards, Jane Mears, Dimitrios Siassakos | Hybrid simulation compared to manikin alone in teaching pelvic examinations: a randomised control trial | 2016 | Lecture  Active Demo | Model | SP | - | - | Comparative: RK+M+/-SP | Formal OSCE station to assess practical and communication skill; self-assessment questionnaire measuring confidence | 2 |
| P31 | Linda E. May, Amber Lievens-Widenski | Pre-clerkship Preparation from Live Patient Practice Pelvic Examinations | 2014 | - | - | GTA | - | - | Singular | Self-assessment questionnaire; student experience | 1 |
| P32 | Lonneke Bokken, Jan-Joost Rethans, Lonneke van Heu | Students' views on the use of real patients and simulated patients in undergraduate medical education | 2009 | - | - | SP | CP | - | Comparative: SP vs CP | 5 group interviews | Insufficient Data |
| P33 | Lorraine Dugoff, Archana Pradhan, Petra Casey, John L. Dalrymple, Jodi F. Abbott, Samantha D. Buery-Joyner, Alice Chuang, Amie J. Cullimore, David A. Forstein, Brittany S. Hampton, Joseph M. Kaczmarczyk, Nadine T. Katz, Francis S. Nuthalapaty, Sarah M. Page-Ramsey, Abigail Wolf and Nancy A. Hueppchen | Pelvic and breast examination skills curricula in United States medical schools: a survey of obstetrics and gynecology clerkship directors | 2016 | Video | Models (with SP)  Model with integrated feedback | SP | CP | - | Combined curriculum review  RK+M+SP+CP | Self-assessment questionnaire | Insufficient Data |
| P34 | Lynn McBain, Susan Pullon, Sue Garrett, Kath Hoare | Genital examination training: assessing the effectiveness of an integrated female and male teaching programme | 2016 | Video | Model | GTA | - | - | Combined RK + M + GTA | Self-assessment using pre- and post-program questionnaires; proficiency, confidence, comfort levels | 1 |
| P35 | Matthias Kiesel, Inga Beyers, Adam Kalisz, Achim Wöckel, Anne Quenzer, Tanja Schlaiß, Christine Wulf and Joachim Diessner | Evaluating the value of a 3D printed model for hands-on training of gynecological pelvic examination | 2022 | - | Model - commercial  Model - 3D printed | - | - | - | Comparative: Commercial vs 3D printed model | Self-assessment; student satisfaction, pelvic anatomy | 2 |
| P36 | Michael Grynberg, Thibault Thubert, Lucie Guilbaud, Anne-Gae¨l Cordier, Sophie Nedellec, Fre´de´ ric Lamazou, Xavier Deffieux | Students' views on the impact of two pedagogical tools for the teaching of breast and pelvic examination techniques (video-clip and training model): A comparative study | 2012 | Lecture  Video | Model | - | CP | - | Combined: RK + M + CP | Self-assessment; satisfaction questionnaire | 2 |
| P37 | Mitric, C; Chow, K; Krishnamurthy, S; Zeng, X Z; L | Impact of a Multidimensional Technical Skills Training Session Before Obstetrics and Gynaecology Clerkship Rotation on Performance and Exposure | 2018 | - | Model | - | CP | - | Comparative: CP +/- M | Formal assessment using post-rotation OSCE; self-assessment using pre- and post-rotation questionnaires measuring confidence, interest, teaching | 2 |
| P38 | Olov Grankvist, Anders D. Olofsson, Rose-Marie Isaksson | Can physicians be replaced with gynecological teaching women to train medical students in their first pelvic examination? A pilot study from Northern Sweden | 2014 | - | - | GTA | - | - | Singular | Self-assessment - interviews before and after teaching session | 1 |
| P39 | Pamela Dull, Danell J Haines | Methods for teaching physical examination skills to medical students | 2003 | - | - | SP | CP | - | Combined curriculum review | Self-assessed (survey for course directors) | Insufficient Data |
| P40 | Paul P Smith, Shelina Choudhury, T Justin Clark | The effectiveness of gynaecological teaching associates in teaching pelvic examination: A systematic review and meta-analysis | 2015 | - | - | GTA | - | - | Literature review | NA | NA |
| P41 | Ronn, R, Smith, W, Magee, B, Hahn, P M, Reid, R L | Can Online Learning Adequately Prepare Medical Students to Undertake a First Female Pelvic Examination? | 2012 | Video  e-learning | - | SP | - | - | Combined: RK + SP | Standardized assessment checklist to check student performance; volunteer assessment of communication and professionalism; and self-assessment of preparedness levels | 2 |
| P42 | S. Abraham, M. Chapman, A. Taylor, A. McBride & C. Boyd | Anxiety and feelings of medical students conducting their first gynecological examination | 2003 | Video | Model | GTA | - | - | Combined:  RK + M + GTA | Self-assessed  anxiety + confidence | 2 |
| P43 | Sally Pickard, Paula Baraitser, Janice Rymer, Johanna Piper | Can gynaecology teaching associates provide high quality effective training for medical students in the United Kingdom? Comparative study | 2003 | Lecture  Active Demo | Model | GTA | - | - | Combined: RK + M + GTA | 2 formal assessments (3rd week & 12th week of reproductive health block) - technical and communication skills | 2 |
| P44 | Sandhya Gadre | Effectiveness of hand made models: An innovative teaching tool for undergraduates in obstetrics and gynaecology | 2019 | Video | Model | - | - | - | Comparative: RK vs M | OSCE stations, perceptions self-assessed | 2 |
| P45 | Sandra E. Carr, MPH, Dianne Carmody, BA | Outcomes of teaching medical students core skills for women's health: The pelvic examination educational program | 2004 | Lecture  e-Learning | Model | GTA | - | - | Combined: RK + M + GTA | Summative end-of-year assessment OSCE station, self-assessed using questionnaire (confidence, anxiety, competence), questionnaire about perception of GTAs | 2 |
| P46 | Sangeeta Jain, Karin Fox, Patricia Van den Berg, Alexandria Hill, Susan Nilsen, Gayle Olson, Bernard Karnath, Ann Frye, Karen Szauter | Simulation Training Impacts Student Confidence and Knowledge for Breast and Pelvic Examination | 2014 | - | Model | SP (GTA) | - | - | Comparative: M+SP+GTA vs GTA | GTAs as assessment only | 2 |
| P47 | Seago, B L, Ketchum, J M, Willett, R M | Pelvic examination skills training with genital teaching associates and a pelvic simulator: does sequence matter? | 2012 | Video | Model | GTA | - | - | Combined: various combinations | Affective self-assessment – via the Fear of Pelvic Examination Scale (F-PEXS), measuring fear, stress | 2 |
| P48 | Shrestha S, Wijma B, Swahnberg K, Siwe K | Learning pelvic examination with professional patients | 2010 | Lecture  Video | Model | GTA | - | - | Combinded: RK + M + GTA | Evaluative talk after teaching session | Insufficient Data |
| P49 | Talles Dias Orsi, Ana Lucia Ribeiro Valadares, Paula Miranda Esteves Orsi, Isabella Miranda Esteves Orsi, Alexandre Sampaio Moura | Treinamento baseado em simulacao para exame fisico pelvico e de mamas: Efeito na ansiedade e autoconfianca dos estudantes de medicina, Simulation-based Training for Pelvic and Breast Physical Examination: Effect on the Anxiety and Self-confidence of Medical Students | 2020 | Lecture  Video | Model | - | - | - | Combined: RK + M | Self-assessment using pre- & post-training questionnaire; satisfaction with session | 2 |
| P50 | Tejal Parikh, Maria Czuzak, Naomi Bui, Corinna Wildner, Bryna Koch, Elizabeth Leko, William Rappaport, Srikar Adhikari, Paul Gordon, Mike Gura, Susan Ellis | Novel Use of Ultrasound to Teach Reproductive System Physical Examination Skills and Pelvic Anatomy | 2018 | Lecture | - | GTA + adj USS | - | - | Combined: RK + GTA&USS | Formal assessment of practical exam; self-assessment using pre-post survey measuring changes in self-reported knowledge, confidence, and anxiety | 2 |
| P51 | Vincent Y. T. Cheung, Yuk Ming Tang, Karen K. L. Chan | Medical students' perception of the application of a virtual reality training model to acquire vaginal examination skills | 2023 | - | Model + VR | - | - | - | Singular | Self-assessed; confidence | 2 |
| P52 | Yolanda Cuñarro-López, Lucia Sánchez Llanos, Ignacio Cueto Hernández, et al | Workshop for Basic Gynaecological Examinations: Improving Medical Student Learning through Clinical Simulation | 2023 | e-Learning | Model | - | - | - | Combined: RK + M | Self-assessed; confidence, satisfaction | 2 |

**Additional Sources from Repeat Search Prior to Publication**

|  |  |  |  |  | **Experiential Knowledge** | | |  |  |  |  |
| --- | --- | --- | --- | --- | --- | --- | --- | --- | --- | --- | --- |
| **No.** | **Author** | **Title** | **Year** | **Received Knowledge (RK)** | **Model (M)** | **Professional Patient (PP)** | **Clinical Practice (CP)** | **Other** | **Approach** | **Evaluation Method** | **Kirkpatrick Level** |
| P53 | Plotzker R.E.  Harmon D.J.  Kanellitsas T.  Klein B.A. | Virtual reality or personal computer-based gynecologic pelvic exam simulation: medical student preferences. | 2025 | Video | VR-Model | - | - | - | Comparative: RK vs. M+VR | Survey  Focus Group | 1 |
| P54 | Shui M.L.  Lypson M.L.  Sewell T.B.  Ratan R.B.  Saab S.S. | Educational experience and ethical tensions in pelvic examination training: a mixed-methods study in the obstetrics and gynecology clerkship. | 2026 | Video | Model | GTA | CP | Feedback | Combined: RK + GTA + M + CP | Survey  OSCE | 2 |
| P55 | Zeng H.  Li M.  Liu N.  Li S. | Mixed reality in medical education: A study on bimanual pelvic examination. | 2025 | Lecture  Active Demo | Model +VR | - | - | - | Comparative: RK + M +/- VR | Survey | 1 |
